# Cytotoxicity, Differentiation, and Biocompatibility of Root-End Filling: A Comprehensive Study

**DOI:** 10.3390/biomimetics8070514

**Published:** 2023-10-29

**Authors:** Ignacio Jimenez-Bueno, Rene Garcia-Contreras, Benjamin Aranda-Herrera, Hiroshi Sakagami, Christian Andrea Lopez-Ayuso, Hiroshi Nakajima, Carlos A. Jurado, Hamid Nurrohman

**Affiliations:** 1 Department of Endodontics, Faculty of Dentistry, Autonomous University State of Mexico (UAEMex), Toluca 50130, State of Mexico, Mexico; endomixijv@yahoo.com.mx; 2 Interdisciplinary Research Laboratory, Nanostructures and Biomaterials Area, National School of Higher Studies (ENES) Leon, National Autonomous University of Mexico (UNAM), Leon 37684, Guanajuato, Mexico; rgarciac@enes.unam.mx (R.G.-C.); lopezayuso@gmail.com (C.A.L.-A.); 3 Meikai University Research Institute of Odontology (M-RIO), Meikai University School of Dentistry, Sakado 350-0283, Saitama, Japan; sakagami@dent.meikai.ac.jp; 4 Division of Dental Biomaterials Science, Department of Restorative and Biomaterials Sciences, Meikai University School of Dentistry, Sakado 350-0283, Saitama, Japan; 5 Department of Prosthodontics, The University of Iowa College of Dentistry and Dental Clinics, Iowa City, IA 52242, USA; 6 Department of Restorative Dentistry & Prosthodontics, University of Texas School of Dentistry, Houston, TX 77054, USA

**Keywords:** MTA, Portland cements, cytotoxicity, death cell, inhibitors

## Abstract

Assessing the biocompatibility of endodontic root-end filling materials through cell line responses is both essential and of utmost importance. This study aimed to the cytotoxicity of the type of cell death through apoptosis and autophagy, and odontoblast cell-like differentiation effects of MTA, zinc oxide–eugenol, and two experimental Portland cements modified with bismuth (Portland Bi) and barium (Portland Ba) on primary cell cultures. Material and methods: The cells corresponded to human periodontal ligament and gingival fibroblasts (HPLF, HGF), human pulp cells (HPC), and human squamous carcinoma cells from three different patients (HSC-2, -3, -4). The cements were inoculcated in different concentrations for cytotoxicity evaluation, DNA fragmentation in electrophoresis, apoptosis caspase activation, and autophagy antigen reaction, odontoblast-like cells were differentiated and tested for mineral deposition. The data were subject to a non-parametric test. Results: All cements caused a dose-dependent reduction in cell viability. Contact with zinc oxide–eugenol induced neither DNA fragmentation nor apoptotic caspase-3 activation and autophagy inhibitors (3-methyladenine, bafilomycin). Portland Bi accelerated significantly (*p* < 0.05) the differentiation of odontoblast-like cells. Within the limitation of this study, it was concluded that Portland cement with bismuth exhibits cytocompatibility and promotes odontoblast-like cell differentiation. This research contributes valuable insights into biocompatibility, suggesting its potential use in endodontic repair and biomimetic remineralization.

## 1. Introduction

Root-end filling materials serve as essential components in endodontic treatments, with their primary objectives rooted in two crucial aspects: establishing an effective seal at the apex of the root and supporting optimal healing processes. These materials play a pivotal role in preventing the escape of harmful bacteria and their byproducts from the root canal system into the surrounding periradicular tissues, safeguarding the patient’s oral health. Furthermore, these materials contribute significantly to the regeneration of critical dental structures, including cementum, periodontal ligament, and alveolar bone, along the resected root-end surface. This regenerative aspect is paramount in ensuring that the patient experiences a full and successful recovery after the endodontic procedure [1].

The diverse range of materials recommended for use as root-end fillings reflects the multifaceted nature of endodontics. These materials include amalgam, resin composite, zinc oxide–eugenol, glass-ionomer cement, polycarboxylate cement, gold foil, and gutta-percha [2,3], each with unique properties and applications. However, it is important to note that, despite the variety of choices available, no single material has emerged as the definitive solution that comprehensively encompasses all the “ideal” characteristics of root-end filling material, as emphasized by Torabinejad et al. in 1993 [4]. In the evaluation of these materials, biocompatibility, and biological properties take center stage. Given their extended contact with the periodontium, ensuring that these materials are biocompatible is paramount for the longevity of treatments. Biocompatibility assessments involve in vitro cytotoxicity screening, wherein various cell types, both primary and transformed, are cultured and studied to assess their reactions to these materials. It is essential to recognize that transformed cells can exhibit distinct biological properties compared to primary human diploid cells, adding a layer of complexity to the evaluation process [5]. Among the notable materials in this context is mineral trioxide aggregate (MTA). This substance has garnered attention for its capacity to seal communication passages between the root canal and the external tooth surface. MTA stands out by promoting the formation of cementum or dentin over its exposed surface, aiding in the restoration and healing of the tooth. Its unique properties make it a valuable asset in endodontic procedures, further highlighting the ever-evolving nature of endodontic materials and techniques [6]. Recent research has shed light on the biocompatibility of root-end filling materials, with specific attention to MTA and its comparative cytotoxicity profile. This valuable investigation has revealed intriguing findings, positioning MTA as a material with lower cytotoxicity when contrasted with zinc oxide–eugenol cement IRM (Dentsply Sirona, Charlotte, NC, USA) and SuperEBA (Harry J. Bosworth, Skokie, IL, USA). However, it is worth noting that MTA still exhibits higher cytotoxicity than amalgam, underscoring the importance of evaluating biocompatibility in endodontic treatments [7].

To delve deeper into this critical aspect of endodontics, our study aimed to assess the biocompatibility effects of MTA, zinc oxide–eugenol endodontic filling material, and two experimental Portland cement modified with bismuth and barium. These evaluations were conducted across six distinct primary cell cultures, covering a spectrum of cell types relevant to endodontics. Our primary cell cultures included normal cells, such as human periodontal ligament fibroblasts (HPLF), human pulp cells (HPC), and human gingival fibroblasts (HGF). Additionally, we incorporated cancer cell lines, specifically human squamous carcinoma cells derived from three different patients (HSC-2, HSC-3, and HSC-4). This comprehensive approach allowed us to gauge the impact of these root-end filling materials across a diverse array of cell types, ensuring a robust assessment of their biocompatibility. Our assessment encompassed several crucial facets, including cytotoxicity analysis, the investigation of DNA fragmentation, the determination of cell death types through apoptosis-caspase activation and autophagy inhibition, and the evaluation of odontoblast cell-like differentiation within HPC cells. The null hypothesis tested was that there was no significant difference in the root-filling materials’ interaction with different cell types and their biological implications for endodontic treatments.

## 2. Materials and Methods

### 2.1. Materials

The subsequent chemicals and reagents were acquired from the enterprises designated accordingly: alpha minimum essential medium (DMEM, GIBCO BRL, Grand Island, NY, USA); fetal bovine serum (FBS, JRH Bioscience, Lenexa, KS, USA); Penicillin Streptomycin (GIBCO BRL, Grand Island, NY, USA); Portland cement (White, Cruz Azul, México) and Portland cement (White, Tolteca, México) modified with bismuth (Portland Bi; Sigma-Aldrich, St. Louis, MO, USA) and barium (Portland Ba; Sigma-Aldrich); MTA (Angelus, Londrina, Brazil), SuperEBA (Harry J. Bosworth, Skokie, IL, USA); MTT [3-(4, 5-dimethyl-thyazol-2-yl)-2, 5-diphenyltetrazolium bromide] (Sigma-Aldrich); and 6-well and 96-well culture plastic dishes and plates were from Becton Dickinson, Franklin Lakes, NJ, USA.

### 2.2. Cells Culture

HPLF, HPC, HGF, and HSC-2, 3, 4 cells were obtained from established stocks of the Department of Diagnostic and Therapeutic Sciences, Meikai University School of Dentistry, stored at −80 °C. Fresh subcultures of cells were grown as adherent cultures in DMEM, enriched with 10% FBS and 2% antibiotics, and maintained at 37 °C in a humidified environment with 5% CO_2_ [8]. For each experiment, cells were rinsed with phosphate-buffered saline (pH 7.4) without calcium and magnesium ions [PBS (-)] and detached using a solution of 0.25% trypsin and 0.025% EDTA-2Na in PBS (-) (GIBCO BRL) [9].

### 2.3. Cytotoxicity of Root-End Filling Materials

Cells were inoculated in 96-microwell plates at 1:3 of 6–8 population doubling level (PDL) for normal cells (HPLF, HGF, HPC). Meanwhile, cancer cells HSC-2, 3, and 4 were inoculated at 5 × 10^4^ cells/mL. Cells were incubated for 48 for complete proliferation and attachment. Cements were added at various concentrations over the cells. Cements were ultrasonically dissolved in distilled water (dH_2_O) except for SuperEBA, blended with dimethyl sulfoxide (DMSO, Wako Pure Chem Co., Tokyo, Japan). Cells and types of cement (0–5 mg/mL) interacted for 24 h at 37 °C in 5% CO_2_, and then the relative viable cell number was determined by the MTT approach. To achieve this, cells were subjected to a 4 h incubation with MTT (0.2 mg/mL) in fresh DMEM. The formazan product generated during the incubation was subsequently dissolved using dimethyl sulfoxide (0.1 mL). The optical absorbance of the resulting lysate was then measured at 540 nm under a microplate reader (Multiskan, Biochromatic, Labsystem, Osaka, Japan). The mean cytotoxic value (IC_50_) was calculated.

### 2.4. Assay for DNA Fragmentation

HPC was seeded at a 1:3 cell density, while HSC-2 cells were plated at a concentration of 3 × 10^4^ cells/mL in 6-well culture plates. Subsequently, a 48 h incubation period allowed for complete cell attachment. Following this incubation period, cells were exposed to varying concentrations of SuperEBA for HSC-2 (ranging from 0 to 250 mg/mL) and HPC (ranging from 0 to 125 mg/mL) over a duration of 6 h. After exposure, cells were subjected to two consecutive washes with PBS (-), and then they were harvested using a rubber policeman while maintaining a cold environment. Cell collection was followed by centrifugation in 1.5 mL microcentrifuge tubes at 20,000× *g*. To isolate biomolecules, the cell pellets underwent lysis in a solution containing 50 μL of lysate buffer composed of 50 mM Tris-HCl (pH 7.8), 10 mM EDTA, and 0.5% (*w*/*v*) sodium N-lauroylsarcosinate. This lysate was further treated with RNase A (0.4 mg/mL) and proteinase K (0.8 mg/mL) for a 2 h incubation period at 50 °C. Subsequently, a NaI solution (50 μL, consisting of 40 mM Tris-HCl, pH 8.0, 7.6 M NaI, and 20 mM EDTA-2Na) was added, followed by ethanol (250 μL). The resulting mixture was then centrifuged for 20 min at 20,000× *g*, leading to the formation of a precipitate. This precipitate was washed with 1 mL of 70% ethanol and subsequently dissolved in TE buffer containing 10 mM Tris-HCl (pH 8.0) and 1 mM EDTA-2Na. Each sample, equivalent to 10–20 μL and corresponding to a cell concentration of 5 × 10^5^ cells/mL, was loaded onto a 2% agarose gel for electrophoresis in TBE buffer composed of 89 mM Tris-HCl, 89 mM boric acid, and 2 mM EDTA-2Na. After electrophoresis, the DNA was visualized through UV irradiation and photographed, as previously described [10]. Furthermore, as a means of positive control, DNA extracted from HL-60 cells undergoing apoptosis induced by UV irradiation (at a rate of 6 J/m^2^/min for 1 min) was simultaneously included in the experiment [11].

### 2.5. Assay for Apoptosis Caspase-3 Activation

HPC cells were initially seeded at a cell density ratio of 1:3, while HSC-2 cells were plated at a concentration of 5 × 10^4^ cells/mL in 80 mm culture dishes. Subsequently, these cells were incubated for 24 h for complete adherence. Following this adherence phase, the cells were subjected to an additional 4 h incubation in a fresh DMEM medium containing the specified concentrations of SuperEBA, which had been previously dissolved in DMSO. SuperEBA concentrations ranged from 0 to 250 mg/mL for HSC-2 cells and from 0 to 125 mg/mL for HPC cells. Subsequently, the cells were subjected to two rinses with PBS (-) and lysed using 200 μL of a lysis solution. Cell lysis was facilitated by scraping the cells with a rubber policeman, and the resulting lysate was transferred to a 1.5 mL microcentrifuge tube. After allowing the lysate to stand for 10 min on ice and centrifuging it for 5 min at 10,000× *g*, the supernatant was carefully collected. In the caspase-3 assay, 50 μL of the lysate solution, which corresponds to 200 μg of protein, was combined with 50 μL of a lysis solution containing caspase-3 substrates, specifically DEVD-pNA (p-nitroanilide) [12]. After a 4 h incubation at 37 °C, the resulting chromophore pNA was quantified using a microplate reader, as described previously, by measuring the absorbance at 405 nm.

### 2.6. Effect of Apoptosis and Autophagy Inhibitors

HPC cells were initially inoculated at a 1:3 density, while HSC-2 cells (6 × 10^4^ cells/mL) were seeded onto a 96-well plate and incubated for 24 h to achieve complete adherence. Subsequently, the cells were preincubated for 60 min with specific compounds: 50 μM of the pan-caspase inhibitor (Z-VAD-FMK, Biomol, Enzo Life Science, Plymouth Meeting, PA, USA), 10 mM of 3-methyl adenine (Sigma-Aldrich), or 100 nM of bafilomycin (BAF) (Wako Pure Chem Co., Tokyo, Japan). SuperEBA (0.5 mg/mL) was added and incubated for 24 h. The viable cell number was assayed by MTT methods at 540 nm [13].

### 2.7. Odontoblast-like Cells Differentiation

To induce odontoblast-like differentiation, HPC (8 PDL) 1 × 10^6^ cells/mL were exposed to an odontoblastic differentiation medium enhanced with 10% FBS, 1% Pen-Strep, dexamethasone at a concentration of 0.05 mM, β-glycerophosphate at 0.5 mM, ascorbic acid at 0.5 mM, transforming growth factor-β3 at 20 ng/mL, and fibroblast growth factor-2 at 5 ng/mL were all sourced from Sigma-Aldrich. Additionally, the medium was enriched with or without Portland Bi (0.5 mg/mL) for a period of 10 days [14]. For the control group, conventional subculture medium cells were utilized. The assessment of mineral deposition activity was carried out using alizarin red (Sigma-Aldrich) [15] and Von Kossa stains [16]. To analyze calcified minerals, the differentiation was interrupted with an alizarin red stain (40 mM) dissolved in NaH_2_PO_3_ (Sigma-Aldrich) at a pH of 4.3 for 5 min. The staining was then washed twice with PBS and fixed with 70% ethanol (*v*/*v*) for 30 min. The culture was rinsed twice with PBS (+), and alizarin red stain was added for 10 min at room temperature. Subsequently, the culture was washed twice with PBS and five times with dH_2_O.

A Von Kossa stain was performed to evaluate calcium deposition by treating the samples with a 5% silver nitrate solution exposed to ultraviolet (UV) light for 20 min, succeeding by a 5% sodium thiosulfate solution for 5 min [16]. Counterstaining was performed using a Hematoxylin solution for 10 min, resulting in black-stained nuclei. The calcium deposition was dissolved with a solution containing 5% isopropanol and 10% acetic acid for 16 h. The absorbance was measured at 550 nm using a 96-microplate reader spectrophotometer. The resulting staining for both methods was photographed for analysis at 40×. All the final five reagents utilized in the experiment were sourced equally and exclusively from Sigma-Aldrich, ensuring consistent and reliable quality throughout the study.

### 2.8. Statistical Analysis

The data were analyzed by calculating the mean, standard deviation, and percentages. To perform non-parametric analysis, we utilized the Statistical Package for the Social Sciences (SPSS) version 19.0 (Chicago, IL, USA). The various cement types were compared using the Kruskal–Wallis test and U Mann–Whitney test, with a predefined significance level set at *p* < 0.05. Experiments corresponded to three samples of three independent experiments (n = 9).

## 3. Results

### 3.1. Cytotoxicity of Root-Ending Cements

**Figure 1:**
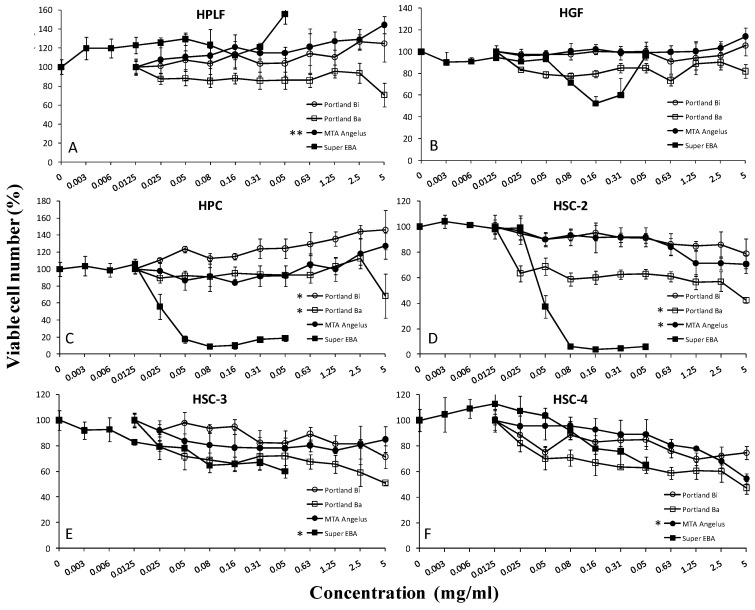
Dose response of Portland Bi, Portland Ba, MTA Angelus, and SuperEBA in cultures with normal cells (**A**) HPLF, (**B**) HGF, (**C**) HPC) at a 1:3 ratio of 6–8 PDL and cancer cells (**D**) HSC-2, (**E**) HSC-3, (**F**) HSC-4 at concentrations of 5 × 10^4^ cell/mL, near 90% confluence and were inoculated in 96-microwell plates and incubated for 48 h with varying concentrations from 0 to 5 mg/mL. The relative viable cell count was assessed using the MTT method. Each value represents the Mean ± SD of triplicate assays (n = 9), 540 nm absorbances. HPLF = human periodontal ligament fibroblast, HGF = human gingival fibroblast, HPC = human pulp cells, HSC = human oral squamous carcinoma cells sourced from three distinct patients (−2, 3, 4). PDL = population doubling level; SD = standard deviation. * *p* < 0.05, ** *p* < 0.01 U Mann–Whitney test.

The relative potency of cytotoxicity of cement was assessed by subjecting the exponentially growing HPLF, HGF, and HPC cells to varying concentrations of each material during the incubation period (Figure 1 and Table 1). All materials produced a dose-dependent minor decline of cell viability without showing growth-stimulating effects at lower concentration ranges (hormetic response). The order of their cytotoxicity for HPLF was in the following order: Portland Ba > Portland Bi > MTA Angelus (*p* = 0.018) > SuperEBA (least toxic) Cytotoxicity of HGF was as follows SuperEBA (most toxic) > Portland Ba > Portland Bi > MTA Angelus (least toxic). The biocompatibility of HPC in contact with cement was superEBA (IC_50_ = 0.05 mg/mL) > Portland Ba (*p* = 0.044) > MTA Angelus > Portland Bi (*p* = 0.026). Cancer cells corresponded to HSC-2: Super EBA (IC_50_= 0.03 mg/mL) > Portland Ba (IC_50_ = 4.69 mg/mL) (*p* = 0.056) > MTA Angelus > Portland Bi, HSC-3: SuperEBA (*p* = 0.049) > Portland Ba > Portland Bi > MTA Angelus, HSC-4: Portland Ba (IC_50_ = 4.2 mg/mL) > Super EBA > MTA Angelus (*p* = 0.033) > Portland Bi (Table 1).

### 3.2. Assay for DNA Fragmentation

**Figure 2:**
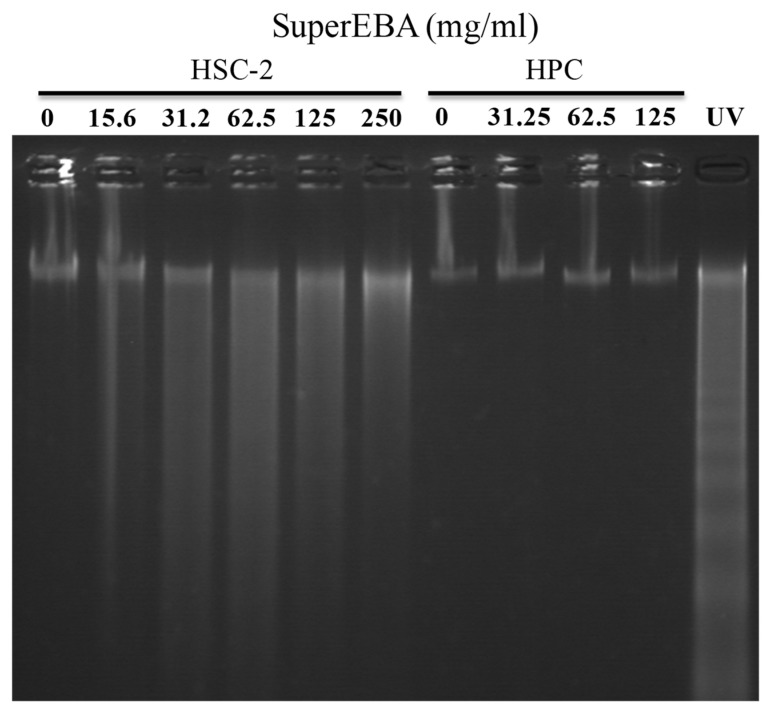
SuperEBA induces internucleosomal DNA fragmentation. HPC were inoculated at 1:3 density and HSC-2, cells (3 × 10^4^ cells/mL) were placed in 6-well plates and incubated for 48 h to achieve full attachment HSC-2 and HPC cells were incubated with the specified concentrations of SuperEBA for 6 h, followed by DNA fragmentation analysis using agarose gel electrophoresis. HL-60 cells exposed to UV irradiation (6 J/m^2^/min, 1 min) were simultaneously included as positive controls. HPC = human pulp cells, HSC-2= human oral squamous carcinoma cells.

Figure 2 illustrates that SuperEBA triggered a smear pattern of DNA fragmentation in HSC-2 cells, ranging from doses of 15.6 to 250 mg/mL. This contrasts with the internucleosomal DNA fragmentation observed in UV-induced apoptotic HPC cells. On the other hand, SuperEBA did not result in either internucleosomal or smear patterns of DNA fragmentation in HPC cells, even at doses ranging from 31.25 to 125 mg/mL.

### 3.3. Assay for Caspase-3 Activation

**Figure 3:**
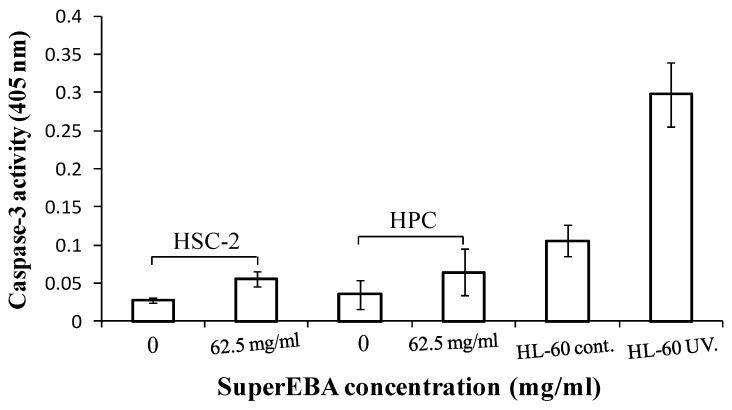
Apoptosis caspase-3 activated. Caspase-3 activation was not induced by SuperEBA in HSC-2 and HPC cultures. After incubating cells with the specified concentrations for 4 h, they were subsequently lysed for the caspase-3 assay, and the results were analyzed by measuring absorbances at 405 nm. Apoptotic HL-60 cells induced by UV irradiation served as the positive control. Each data point represents the mean ± SD derived from triplicate assays (n = 9). HL-60 cells induced by UV irradiation at a rate of 6 J/m^2^/min, 1 min as additional positive controls. HPC = human pulp cells, HSC-2= human oral squamous carcinoma cells.

Figure 3 depicts that SuperEBA at a concentration of 62.5 mg/mL did not induce caspase-3 activation, a process known to stimulate caspase-activated DNase, also known as ‘CAD’ [17] in HSC-2 and HPC cells, contrasting with the observation during apoptosis in UV-induced HL-60 cells.

### 3.4. Effect of Apoptosis and Autophagy Inhibitors

**Figure 4:**
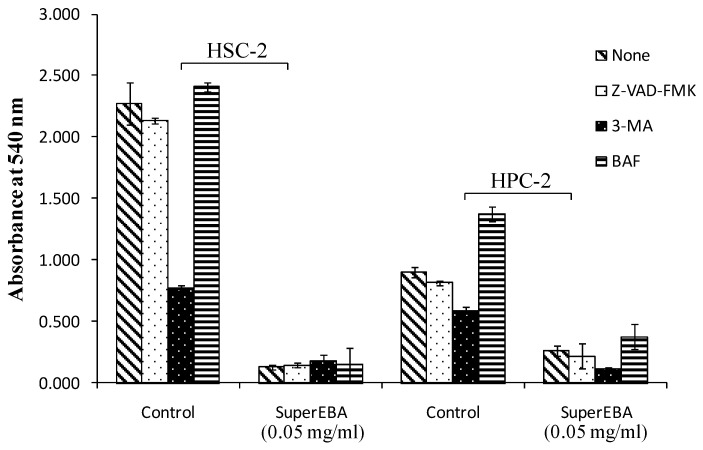
The impact of various inhibitors on SuperEBA-induced cytotoxicity was examined. Prior to exposure, cells were pre-incubated for 1 h under four distinct conditions: untreated (control), treated with caspase-3 inhibitor (Z-VAD-FMK, 50 μM), exposed to 3-methyl adenine (3-MA, 10 mM), or treated with bafilomycin (BAF, 100 nM) and then SuperEBA added. Subsequently, they were incubated 24 h. The viable cell number was determined by MTT assay. Each data point represents the mean ± SD derived from triplicate assays (n = 9). HPC = human pulp cells, HSC-2= human oral squamous carcinoma cells.

In the context of HSC-2 cell culture (Figure 4), the caspase inhibitor 3-MA had a singularly adverse effect on cell viability compared to Z-VAD-FMK and BAF. Conversely, Z-VAD-FMK and BAF did not exhibit a significant difference compared to cells without a caspase inhibitor. In HPC culture, 3-MA alone reduced cell viability, while BAF increased it. However, preincubation of SuperEBA at 0.05 mg/mL with caspase inhibitors did not protect HSC-2 cells from induced cytotoxicity. These inhibitors also reduced cell viability induced by 0.05 mg/mL concentrations of SuperEBA in HPC culture.

### 3.5. Odontoblast-like Cells Differentiation HPC

**Figure 5:**
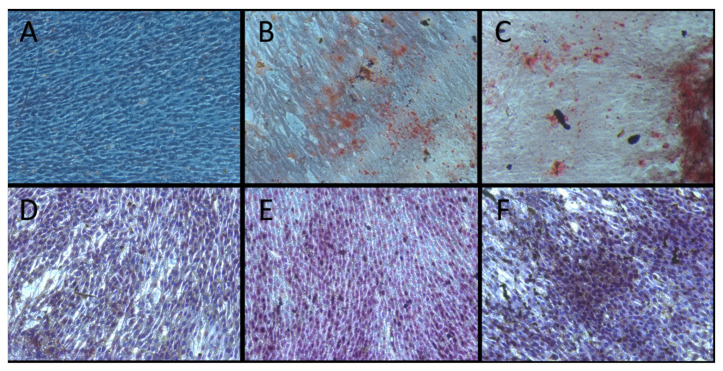
HPC differentiation into odontoblast-like cells was carried out in a culture, both with and without the presence of Portland Bi, over a 10-day period. HPC at 90% confluence and 8 PDL were subcultured and then differentiated to odontoblast-like cells. The evaluation of differentiation was performed using alizarin red stain (**A**–**C**) and Von Kossa stain (**D**–**F**). (**A**,**D**) HPC controls were exposed to a standard culture medium, and in this context, the HPC did not display any evidence of matrix biomineralization. (**B**,**E**) HPC was treated with the odontoblastic medium without Portland Bi. (**C**,**F**) HPC was treated with odontoblastic medium containing 0.05 mg/mL, exhibiting more matrix biomineralization. The microphotographs were captured using a light microscope at 20×. HPC = human pulp cells.

The staining results of alizarin red and Von Kossa are depicted in Figure 5. No deposition of minerals on the cell surface was discernible in the control group (0.08 ± 0.002 abs), where standard growth media was employed (Figure 5A,D). However, in the medium enriched with differentiation growth media (0.08 ± 0.002 abs) (Figure 5B,E) and with Portland Bi (C, F), a statistically significant increase (0.09 ± 0.001 abs, *p* < 0.05) in matrix biomineralization and the presence of calcified nodes were evident compared to the control group. Although the cell culture period was relatively short, it led to limited mineral deposition, and mineral deposits were formed across the cytoplasm and on the cell surface.

## 4. Discussion

This study was designed to evaluate the effect of MTA Angelus, SuperEBA, and two Portland cements on six-line cell viability. Instead of counting the number of cells prior to and after exposure to materials, we performed an MTT technique that allowed us to quantify the percentage of cells undergoing programmed cell death and to determine the percentage. The chosen experimental approach provided us with a more comprehensive insight into how dental materials impact the survival and mitotic activity of pulp cells.

### 4.1. Cytotoxicity of Root-Ending Cements

MTA Angelus and two different Portland cements exhibited significantly lower cytotoxicity compared to SuperEBA. These findings align with previous studies that highlight the biocompatibility of MTA [9,18,19]. There were no statistically significant variations observed in the extent of cytotoxicity between the two Portland cement brands, and it was noted that cytotoxicity decreased progressively over time. The results of our study indicate that MTA Angelus exhibited minimal cytotoxic effects on pulp cells (RPC-C2A). These findings are consistent with prior research supporting the favorable biocompatibility of MTA Angelus in endothelial cells and macrophages [20,21,22,23].

SuperEBA cement contains a powdered component consisting of zinc oxide (65%), fused quartz or alumina (20–35%), and hydrogenated resin (6%). Its liquid component comprises 63% ethoxy benzoic acid (EBA) and 37% eugenol. Zinc oxide–eugenol cements are known to have the potential to induce inflammatory reactions in tissues, primarily due to the presence of free eugenol. Many studies have reported the cytotoxic effects of SuperEBA, which can be attributed to its eugenol content. Eugenol is commonly used as an antimicrobial and anti-inflammatory agent; however, previous in vitro and in vivo investigations have revealed its toxic effects [24,25,26,27,28,29].

The development of novel materials aimed at enhancing cellular responses in the periapical region following endodontic treatment has been an ongoing pursuit. Some recent materials designed for root-end applications, such as Ceraputty endodontic cement containing zirconium dioxide, tricalcium silicate, dicalcium silicate, and tricalcium aluminate, have undergone in vitro testing. However, their evaluation using human periodontal ligament stem cells (hPDLSCs) has revealed cytotoxic effects, even at dilutions of 1:2, 1:4, and undiluted, in comparison to other cement types like Biodentine or Endosequence BC RRM Putty [30]. Despite the introduction of these new materials, further research is necessary to comprehend the cytotoxicity and periapical inflammation responses associated with the various endodontic cements available today.

### 4.2. Assay for DNA Fragmentation

DNA fragmentation assays are techniques used in molecular biology to evaluate the integrity of DNA; in materials science, it allows us to determine if the DNA in a cell or tissue has experienced fragmentation when exposed to a specific material, which may be indicative of cell damage [31]. In the case of root canal sealers, they may release degradation products that encounter periodontal tissue and may cause some type of DNA damage. In this regard, some studies have analyzed endodontic cements based on calcium hydroxide, zinc oxide, eugenol, and epoxy resin without identifying DNA damage, reporting only dose-dependent cytotoxicity [32]. In this study, SuperEBA cement caused irreversible cell death mainly in HPC and HSC-2, identifying them as the most sensitive cells, so the cell fragmentation assay was developed in these cell lines, finding solely a pattern of DNA fragmentation in HSC-2 cells, which may be related to cell apoptotic processes [31].

### 4.3. Caspase-3 Activation and Autophagy

Caspase-3 is a protease closely related to apoptosis, cell growth, and differentiation. While zinc oxide and eugenol-based cement, along with mineral trioxide aggregates, are generally considered to have acceptable biocompatibility, they are known to be highly technique sensitive and can be difficult to mix and handle effectively. Previous research has consistently reported that eugenol has inhibitory effects on critical cellular processes such as cell migration, prostaglandin synthesis, cellular respiration, and mitochondrial activity [26,27,28]. Furthermore, eugenol has been found to induce alterations in the cell membrane [24] and trigger the stimulation of neutrophils [33,34,35].

### 4.4. Odontoblast-Like Cells Differentiation

Portland cement primarily consists of alite, belite, aluminate, and ferrite. It is classified into five types based on varying compound proportions: Type I is common and has high tricalcium silicate, while Type II has low tricalcium aluminate. Type III has fine particles and high early strength, while Types IV and V contain less tricalcium aluminate. Studies show Portland cement’s potential for biomineralization and bone remodeling markers in various contexts, including dental applications and osteosarcoma cells. Additionally, it enhances odontoblastic differentiation and biomineralization gene expression, promoting dentinogenesis and cell proliferation in dental pulp stem cells when pre-treated with pure Portland cement [36,37,38,39,40]. As a result, bismuth-based compounds have been widely used in clinics as radiopacifiers for over a decade [33,34,35]. The bioactivity of bismuth-based compounds has been documented through its ability to enhance alkaline phosphatase activity in human osteoblast-like cells [41]. This observed bioactivity has a direct correlation with the increased formation of calcified deposits during cellular differentiation, a result that strongly corroborates our own research findings. Moreover, when combined with Portland cement, these bismuth-based compounds display a notable absence of cytotoxic effects. Studies have explored implantation methods, including scaffold embedding in platelet-rich plasma (PRP), using an organotypic model with human root segments. These tests, with or without bioactive cements like ProRoot MTA or Biodentine, revealed the regenerative potential of MTA when in contact with apical papilla stem cells (SCAPs). MTA shows promise as an alternative to other dental stem cells (DSCs) for human tooth root microenvironment regeneration [42].

The importance of this study is underscored by the absence of comprehensive research on the potential use of Portland cement in pulp regeneration. Additionally, there is a need for an in-depth examination of the effects of zinc oxide–eugenol cement on different types of normal and cancer cells to determine the specific cell death mechanisms induced in culture.

Nevertheless, limitations of this study include the use of cell cultures, which may not fully replicate the complex in vivo environment of human tissues. Additionally, while a diverse range of cell types was assessed, the study did not account for potential variations within each cell type. Furthermore, the in vitro nature of the study may not capture the dynamic interactions that occur in real clinical scenarios.

## 5. Conclusions

Within the limitations of this study, it was concluded that SuperEBA exhibited the highest cytotoxicity among the tested materials, with varying degrees of toxicity observed in different cell lines. In contrast, MTA Angelus and the two types of Portland cement demonstrated significantly lower cytotoxicity compared to SuperEBA. Notably, no statistically significant differences in cytotoxicity were observed between the two experimental Portland cements. SuperEBA induced a distinct smear pattern of DNA fragmentation in HSC-2 cancer cells, suggesting its potential to activate programmed cell death pathways in cancer cells. However, MTA Angelus did not induce such DNA fragmentation in HPC cells, indicating a differential response between cancer and normal pulp cells. SuperEBA did not activate caspase-3 in either HSC-2 or HPC cell cultures. This suggests that the cytotoxicity of SuperEBA in these cells may not be mediated through caspase-3 activation, a key enzyme associated with apoptosis.

These findings support the notion that MTA Angelus and Portland cement are safer options in terms of biocompatibility compared to SuperEBA. However, it is important to note that cytotoxicity may vary depending on the cell type and material concentration. Portland cement with bismuth exhibits cytocompatibility and promotes odontoblast-like cell differentiation. Overall, these findings underscore the importance of selecting root-end filling materials carefully, taking into consideration their cytotoxicity profile and potential impact on different cell types. MTA Angelus and certain Portland cement formulations, particularly those modified with bismuth, appear to offer safer options in terms of biocompatibility, making them valuable choices for endodontic procedures. However, it is essential to acknowledge that the cytotoxicity of these materials can vary depending on cell type and material concentration. Further research and clinical studies are warranted to validate these findings and guide clinical decision making in endodontics.

## Figures and Tables

**Table 1:** Mean cytotoxic (IC_50_) activity of cements. Most toxic to least toxic up to down I = Portland Ba, II = Portland Bi, III = MTA Angelus, IV = SuperEBA.

Cell Line	Endodontic Cements	Toxicity IC_50_ = mg/mL	Kruskal–Wallis
Normal Cells			
HPLF	I	ND	-
	II	ND	-
	III	ND	*p* = 0.018
	IV	ND	-
HGF	IV	ND	-
	I	ND	-
	II	ND	-
	III	ND	-
HPC	IV	0.05 mg/mL	-
	II	ND	*p* = 0.026
	III	ND	-
	I	ND	*p* = 0.044
Cancer Cells			
HSC-2	IV	0.03 mg/mL	-
	I	4.69 mg/mL	*p* = 0.056
	III	ND	-
	II	ND	-
HSC-3	IV	ND	*p* = 0.049
	I	ND	-
	II	ND	-
	III	ND	-
HSC-4	I	4.2 mg/mL	-
	IV	ND	-
	III	ND	*p* = 0.033
	II	ND	-

IC_50_ = half maximal inhibitory concentration. ND = non-determined.

## Data Availability

Not applicable.

## References

[1] Kumar N., Maher N., Amin F., Ghabbani H., Zafar M.S., Rodríguez-Lozano F.J., Oñate-Sánchez R.E. (2022). Biomimetic Approaches in Clinical Endodontics. Biomimetics.

[2] Gutmann J.L. (2014). Surgical endodontics: Past, present, and future. Endod. Topics..

[3] Friedman S. (1991). Retrograde approach in endodontic therapy. Dent. Traum..

[4] Torabinejad M., Watson T.F., Pitt Ford T.R. (1993). Sealing ability of a mineral trioxide aggregate when used as a root end filling material. J. Endod..

[5] Yesilsoy C., Feigal R.J. (1985). Effects of endodontic materials on cell viability across standard pore size filters. J. Endod..

[6] Torabinejad M., Hong C.U., Lee S.J., Pitt Ford T.R. (1995). Investigation of mineral trioxide aggregate for root-end filling in dogs. J. Endod..

[7] Torabinejad M.C., Hong C.U., Pitt Ford T.R., Kettering J.D. (1995). Cytotoxicity of four root end filling materials. J. Endod..

[8] Garcia-Contreras R., Chavez-Granados P.A., Jurado C.A., Aranda-Herrera B., Afrashtehfar K.I., Nurrohman H. (2023). Natural Bioactive Epigallocatechin-Gallate Promote Bond Strength and Differentiation of Odontoblast-like Cells. Biomimetics.

[9] Koulaouzidou E.A., Papazisis K.T., Economides N.A., Beltes P., Kortsaris A.H. (2005). Antiproliferative Effect of Mineral Trioxide Aggregate, Zinc Oxide-Eugenol Cement, and Glass-Ionomer Cement Against Three Fibroblastic Cell Lines. J. Endod..

[10] Green M.R., Sambrook J. (2019). Analysis of DNA by Agarose Gel Electrophoresis. Cold Spring. Harb. Protoc..

[11] Salucci S., Burattini S., Battistelli M., Baldassarri V., Maltarello M.C., Falcieri E. (2012). Ultraviolet B (UVB) Irradiation-Induced Apoptosis in Various Cell Lineages in Vitro. Int. J. Mol. Sci..

[12] Guerlava P., Izac V., Tholozan J.L. (1998). Comparison of Different Methods of Cell Lysis and Protein Measurements in Clostridium perfringens: Application to the Cell Volume Determination. Curr. Microbiol..

[13] Takano S., Shiomoto S., Inoue K.Y., Ino K., Shiku H., Matsue T. (2014). Electrochemical Approach for the Development of a Simple Method for Detecting Cell Apoptosis Based on Caspase-3 Activity. Anal. Chem..

[14] Yang Y., Zhao Y., Liu X., Chen Y., Lui P., Zhao L. (2017). Effect of SOX_2_ on odontoblast differentiation of dental pulp stem cells. Mol. Med. Rep..

[15] Gregory C.A., Grady Gunn W., Peister A., Prockop D. (2004). An Alizarin red-based assay of mineralization by adherent cells in culture: Comparison with cetylpyridinium chloride extraction. Anal. Biochem..

[16] Rungby J., Kassem M., Eriksen E.F., Danscher G. (1993). The von Kossa reaction for calcium deposits: Silver lactate staining increases sensitivity and reduces background. Histochem. J..

[17] Larsen B.D., Rampalli S., Burns L.E., Brunette S., Dilworth F.J., Megeney L.A. (2010). Caspase 3/caspase-activated DNase promote cell differentiation by inducing DNA strand breaks. Proc. Natl. Acad. Sci. USA.

[18] Osorio R.M., Hefti A., Vertucci F.J., Shawley A.L. (1998). Cytotoxicity of endodontic materials. J. Endod..

[19] Karimjee C.K., Koka S., Rallis D.M., Gound T.G. (2006). Cellular toxicity of mineral trioxide aggregate mixed with an alternative delivery vehicle. Oral. Surg. Oral. Med. Oral. Pathol. Oral. Radiol. Endod..

[20] De Deus G., Ximenes R., Gurgel-Filho E.D., Plotkowski M.C., Coutinho-Filho T. (2005). Cytotoxicity of MTA and Portland cement on human ECV 304 endothelial cells. Int. Endod. J..

[21] Rezende T.M.B., Vargas D.L., Cardoso F.P., Sobrinho A.P., Vieira L.Q. (2005). Effect of mineral trioxide aggregate on cytokine production by peritoneal macrophages. Int. Endod. J..

[22] Tomás-Catalá C.J., Collado-González M., García-Bernal D., Oñate-Sánchez R.E., Forner L., Llena C., Lozano A., Castelo-Baz P., Moraleda J.M., Rodríguez-Lozano F.J. (2017). Comparative analysis of the biological effects of the endodontic bioactive cements MTA-Angelus, MTA Repair HP and NeoMTA Plus on human dental pulp stem cells. Int. Endod. J..

[23] Ferreira C.M.A., Sassone L.M., Gonçalves A.S., de Carvalho J.J., Tomás-Catalá C.J., García-Bernal D., Oñate-Sánchez R.E., Rodríguez-Lozano F.J., Silva E.J.N.L. (2019). Physicochemical, cytotoxicity and in vivo biocompatibility of a high-plasticity calcium-silicate based material. Sci. Rep..

[24] Kasugai S., Hasegawa N., Ogura H. (1991). Application of the MTT Colorimetric Assay to Measure Cytotoxic Effects of Phenolic Compounds on Established Rat Dental Pulp Cells. J. Dent. Res..

[25] Ho Y.-C., Huang F.-M., Chang Y.-C. (2006). Mechanisms of cytotoxicity of eugenol in human osteoblastic cells in vitro. Int. Endod. J..

[26] Fujisawa S., Kadoma Y., Komoda Y. (1988). 1H and 13C NMR Studies of the Interaction of Eugenol, Phenol, and Triethyleneglycol Dimethacrylate with Phospholipid Liposomes as a Model System for Odontoblast Membranes. J. Dent. Res..

[27] Hume W.R. (2007). In vitro studies on the local pharmacodynamics, pharmacology and toxicology of eugenol and zinc oxide-eugenol. Int. Endod. J..

[28] Gerosa R., Borin M., Menegazzi G., Puttini M., Cavalleri G. (1996). In vitro evaluation of the cytotoxicity of pure eugenol. J. Endod..

[29] Mcdonald J.W., Heffner J.E. (1991). Eugenol Causes Oxidant-mediated Edema in Isolated Perfused Rabbit Lungs. Am. Rev. Respir. Dis..

[30] López-García S., Rodríguez-Lozano F.J., Sanz J.L., Forner L., Pecci-Lloret M., Lozano A., Murcia L., Sánchez-Bautista S., Oñate-Sánchez R.E. (2023). Biological properties of Ceraputty as a retrograde filling material: An in vitro study on hPDLSCs. Clin. Oral. Investig..

[31] Garcia-Contreras R., Sakagami H., Nakajima H., Shimada J. (2010). Type of cell death induced by various metal cations in cultured human gingival fibroblasts. In Vivo.

[32] Huang T.H., Ding S.J., Hsu T.Z., Lee Z.D., Kao C.T. (2004). Root canal sealers induce cytotoxicity and necrosis. J. Mater. Sci. Mater. Med..

[33] Chen F., Liu C., Mao Y. (2010). Bismuth-doped injectable calcium phosphate cement with improved radiopacity and potent antimicrobial activity for root canal filling. Acta. Biomater..

[34] Johnson B.R. (1999). Considerations in the selection of a root-end filling material. Oral. Surg. Oral. Med. Oral. Pathol. Oral. Radiol. Endod..

[35] Lei X., Wang J., Chen J., Gao J., Zhang J., Zhao Q., Tang J., Fang W., Li J., Li Y. (2021). The in vitro evaluation of antibacterial efficacy optimized with cellular apoptosis on multi-functional polyurethane sealers for the root canal treatment. J. Mater. Chem. B.

[36] Viola N.V., Guerreiro-Tanomaru J.M., da Silva G.F., Sasso-Cerri E., Tanomaru-Filho M., Cerri P.S. (2012). Biocompatibility of an experimental MTA sealer implanted in the rat subcutaneous: Quantitative and immunohistochemical evaluation. J. Biomed. Mater. Res. B Appl. Biomater..

[37] Min K., Kim H., Park H.J., Pi S.H., Hong C.U., Kim E.C. (2007). Human Pulp Cells Response to Portland Cement In Vitro. J. Endod..

[38] Lee S.-K., Lee S.K., Lee S.I., Park J.H., Jang J.H., Kim H.W., Kim E.C. (2010). Effect of Calcium Phosphate Cements on Growth and Odontoblastic Differentiation in Human Dental Pulp Cells. J. Endod..

[39] Min K.S., Lee S.I., Lee Y., Kim E.C. (2009). Effect of radiopaque Portland cement on mineralization in human dental pulp cells. Oral. Surg. Oral. Med. Oral. Pathol. Oral. Radiol. Endod..

[40] Dreger L.A., Felippe W.T., Reyes-Carmona J.F., Felippe G.S., Bortoluzzi E.A., Felippe M.C. (2012). Mineral Trioxide Aggregate and Portland Cement Promote Biomineralization In Vivo. J. Endod..

[41] Chen Y.-Z., Lü X.-Y., Liu G.-D. (2018). Effects of different radio-opacifying agents on physicochemical and biological properties of a novel root-end filling material. PLoS ONE.

[42] Sequeira D.B., Oliveira A.R., Seabra C.M., Palma P.J., Ramos C., Figueiredo M.H., Santos A.C., Cardoso A.L., Peça J., Santos J.M. (2021). Regeneration of pulp-dentin complex using human stem cells of the apical papilla: In vivo interaction with two bioactive materials. Clin. Oral. Investig..

